# 

**Published:** 1894-12-08

**Authors:** 


PRICE TWOPENCE.

No. 428, Vol. XVII.—Dec. 8th, 1894.

[Registered at the General Post Office as a Newspaper.]

WITH EXTRA NURSING SUPPLEMENT
Per Annum, 10s. 6d., post free.
Monthly Parts, 6d.; post free, 8d.
Ditto per Annum, 8s. 6d. post free.

### THE HOSPITAL

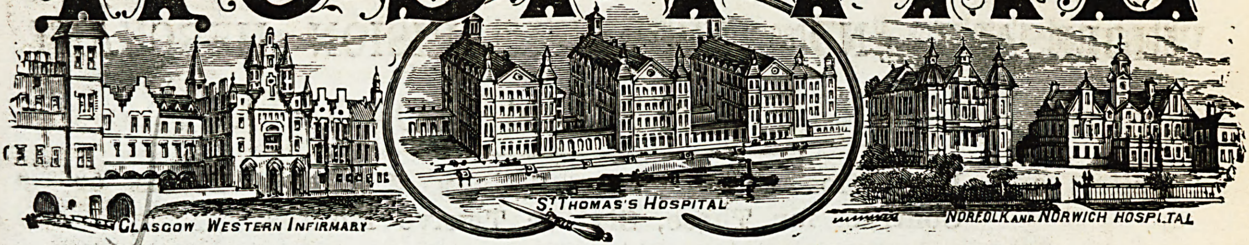

No. 428. Vol. XVII. WITH EXTRA NURSING SUPPLEMENT. Saturday, Dec. 8th, 1894.

